# Cataract as It Is Met with in Ordinary Practice

**Published:** 1895-05-25

**Authors:** R. Lawford Knaggs

**Affiliations:** Assistant Surgeon to the Leeds General Infirmary, and Ophthalmic Surgeon to the Leeds Public Dispensary


### Medical Progress and Hospital Clinics.

[*The Editor will be glad to receive offers of co-operation and contributions from members of the profession. All letters should be addressed to* The Editor, The Lodge, Porchester Square, London, W.]

### CATARACT AS IT IS MET WITH IN ORDINARY PRACTICE.

By R. Lawford Knaggs, Assistant Surgeon to the Leeds General Infirmary, and Ophthalmic Surgeon to the Leeds Public Dispensary.

(*Continued from page* 114.)

The additional gravity which invests the case when the injury is situated within the dangerous ciliary zone of the sclerotic, which surrounds the cornea, or when there is reason to fear that a foreign body remains within the eye, need not now occupy our time, but at least requires recognition.

When the lens matter has been completely absorbed, and the dangers of iritis and glaucoma have been successfully avoided, a membrane more or less opaque separates the vitreous from the anterior chamber, and remains an obstruction to vision; and before the treatment can be considered to have been brought to a satisfactory conclusion, a gap will have to be made in the centre of this membrane. Finally, after this, as after the removal of any form of cataract, the eye requires a strong convex lens before it can obtain distinct vision.

When no sight can be restored by the removal of a cataract, no operation should be performed, unless it is desired for the sake of appearance. Those signs, therefore, by which a healthy condition of the retina and of the other structures behind an opaque lens can be recognised are important. The easiest to elicit is contraction of the pupil to light, but it is necessary to close the other eye carefully to be sure that the contraction is the result of direct stimulation of the retina of the eye that is being tested, and not a consensual contraction dependent upon stimulation of the other retina. Better still is the information obtained by holding a lighted candle at different places in front of the affected eye, within the area of its probable field. This should be done in the dark. If the patient can accurately locate its varying positions, it shows that the retina is functionally active over a wide area. This capability is called "projection," and if, in a case of complete cataract, the pupil is responsive to light, and "the projection" is good, there is every reason to hope that a useful eye will be obtained by the removal of the lens. When perception of light is absent, the eye is blind and to operate is useless. There are of course intermediate cases where "projection" is more or less impaired, and in such, where only an imperfect eye can at the best be hoped for, the propriety of operating will have to be decided by the circumstances of the case.

The frequency of the association of cataract with such an important constitutional disease as diabetes invests it with a special interest for those who see much medical practice. Sometimes a glycosuria, which may disappear under the simplest treatment, is found to exist when the patient first comes for advice about his sight. In other cases the glycosuria is marked and persistent, though the amount o urine is not excessive, whilst in others there are the usual well marked diabetic symptoms. A feature of the diabetic cataract is the rapidity of its formation. It is possible that in the early stage, at least in some cases, the opacity is not due to degeneration of lens fibres, for there are a few well authenticated instances in which cataracts formed during diabetes have completely and rapidly cleared up, with perfect restoration of sight, as the constitutional state improved under treatment, and in one case the opacity returned when the diabetic condition relapsed.

We cannot do better than consider in the short space still at our disposal a question of such practical importance as the bearing that this constitutional affection has upon the question of operative interference.

There is no doubt that many of these cases do very well, and that a temporary glycosuria—and even a permanent one, in which there is no associated polyuria—is no bar to a successful issue, and need not lead to any hesitation in recommending operation. In those cases where the diabetes has assumed a chronic form, and is capable of being ameliorated by suitable treatment, favourable results may reasonably be hoped for. But when the disease is acute, when treatment fails to keep it in check, and when the patient, in spite of every precaution, is going down hill, to advise operation is to undertake a grave responsibility.

Though even in this state the healing of the wound may present no difficulty, yet the anxiety and mental disturbance inseparable from an operation of such importance cannot fail to exert a prejudicial influence upon the course of the disease. In the Guy's Hospital Reports (Vol. XLIX., 1892), Messrs. Bellingham Smith and H. E. Durham, in endeavouring to show that operation in diabetic cataract is not always free from risk, quote three cases which terminated in diabetic coma, one on the eighth day, another on the day of operation, and the third on the third day; and though in the first of these death was attributed to worry, quite apart from the operation, yet they are sufficient to show that the effect that may be produced by operative procedures upon the progress of the constitutional affection is a matter for serious consideration in any particular case.

